# “Traditional” Sol-Gel Chemistry as a Powerful Tool for the Preparation of Supported Metal and Metal Oxide Catalysts

**DOI:** 10.3390/ma12040668

**Published:** 2019-02-23

**Authors:** Serena Esposito

**Affiliations:** Department of Civil and Mechanical Engineering and INSTM Research Unit, Università degli Studi di Cassino e del Lazio Meridionale, Via G. Di Biasio 43, 03043 Cassino FR, Italy; s.esposito@unicas.it

**Keywords:** sol-gel chemistry, metal oxide, heterogeneous catalysts, dispersed metal catalysts, transition metal-based catalysts

## Abstract

The sol-gel method is an attractive synthetic approach in the design of advanced catalytic formulations that are based on metal and metal oxide with high degree of structural and compositional homogeneity. Nowadays, though it originated with the hydrolysis and condensation of metal alkoxides, sol-gel chemistry gathers plenty of fascinating strategies to prepare materials from solution state precursors. Low temperature chemistry, reproducibility, and high surface to volume ratios of obtained products are features that add merit to this technology. The development of different and fascinating procedure was fostered by the availability of new molecular precursors, chelating agents and templates, with the great advantage of tailoring the physico-chemical properties of the materials through the manipulation of the synthesis conditions. The aim of this review is to present an overview of the “traditional” sol-gel synthesis of tailored and multifunctional inorganic materials and their application in the main domain of heterogeneous catalysis. One of the main achievements is to stress the versatility of sol-gel preparation by highlighting its advantage over other preparation methods through some specific examples of the synthesis of catalysts.

## 1. Introduction

Because of their potential applications in most of the industrial chemical processes, metal oxides are of tremendous current interest to scientists and engineers. In the heterogeneous catalysis, oxides-based materials have a prominent role, since they cover the majority of catalyst families used industrially, such as silica, alumina, clays, zeolites, TiO_2_, ZnO, ZrO_2_, porous and mesoporous metal oxides, multicomponent mixed oxides, polyoxometallates (POMs), the phosphates family, and the perovskites [1,2,3,4,5,6,7,8,9,10].

The main issue in the preparation of catalytic materials that can be employed on an industrial scale is to obtain a product with high activity, selectivity, and stability. The need for successful and cost-effective processes spurred the development of versatile methods to produce catalytic materials with improved functionality. The synthesis strategies are devoted to address some main points since the conversion of reagents into product takes place over surface ensembles of atoms: increase the number of sites, control the nature of active sites, and enable the accessibility of the reactant to the active sites [4,5,6,7,8,9,10]. These requirements are generally obtained by deposition of the active component on the surface of a support, which is able not only to disperse the metal, but also to increase its thermal stability and hence the catalyst life [1,11,12]. Mixed-oxide catalysts and supported metal oxide catalysts are commonly prepared by post synthesis methods: impregnation or ion-exchange, coprecipitation and deposition precipitation procedures [13].

Originally used for synthesizing high-quality SiO_2_, the sol-gel process has emerged as a standard production technique for engineering materials [4,5,6,14]. Unlike the more conventional methods, the sol-gel technique allows for preparing porous materials in a “one-pot” with a homogeneous distribution of components on the atomic scale through a technology of low temperature synthesis and with full control of the finite product microstructure. The sol-gel chemistry involves two distinct phases: solution and gelation: a sol is a colloidal suspension of solid particles, whereas a gel is an interconnected network of solid phase particles that form a continuous entity throughout a secondary, usually liquid, phase [15,16,17]. The advantages of sol-gel methods include: high yield, low operation temperatures, and low production costs. Even more, the sol-gel synthesis resulted in possessing unique features, namely the possibility of control over the physico–chemical properties of the resulting compounds through a careful variation of the parameters affecting the various synthesis steps.

The sol-gel process is generally considered as “soft chemistry” in contrast to more classical industrial techniques for glass and ceramic manufacturing, which require very high temperatures [4]. Nowadays, the term ‘sol-gel’ is used more broadly as covering the synthesis of solid materials, such as metal oxides, from solution-state precursors. The development of technologically advanced synthesis routes that overcome the obstacles that are encountered in the most traditional procedures allowed the sol-gel technique to become a unique tool in the design of metal oxides with controlled architecture. Besides the periodic mesoporous silicas obtained taking advantage of self-assembly of the structure directing agent, there are other mesoporous oxides that have made the templating-assisted method popular in the preparation of catalysts [18,19,20,21,22,23,24,25]. Titania has dominant role in the environmental pollutant cleanup and photocatalysis applications, different shapes and reactivity were obtained by template-assisted sol-gel synthesis, making it competitive with the commercial Degussa P-25. Moreover, the possibility to enhance the intrinsic properties and tune nanoparticles morphology and size widened the fields of application [18,19,20,21].

The use of sol-gel chemistry to synthesize semiconductor oxide nanostructures is widespread; ZnO, WO_3_, and SnO_2_ are currently receiving great attention for the photocatalytic water splitting [22,23]. Water splitting has been considered as the most important option for the environmentally friendly production of hydrogen. The added value of sol-gel comes from the possibility of employing the homogeneous solution that was obtained before the gelation to prepare thin films by means of spin and dip coating [22,23].

The use of high thermal stability supports with a low concentration of Lewis acid sites and/or the presence of basic sites, such as ZrO_2_, resulted in enhanced activities in many reactions, e.g., the CO_2_ reforming of methane [24,25]. Montoya et al. [24] reported that the sol-gel synthesis guarantees the homogeneous incorporation of high amounts of Ni and Ce to the t-ZrO_2_ lattice. The incorporation of CeO_2_ both promoted the Ni surface dispersion and NiO reducibility. Cerium oxide also contributed to limiting the tetragonal-to-monoclinic phase transformation. Zirconia oxide is also added to improve the textural properties and the oxygen storage capacity of CeO_2_, widely used in automotive exhaust control. Avoiding segregation of the cerium or zirconium oxides to obtain a definite crystalline structure is crucial for the improvement of catalytic activity. Alifanti et al. [25] showed to which extent this phenomenon can be controlled by the synthesis route reporting a comparison between citrate and the sol-gel methods.

Whereas, recent reviews on sol-gel preparation of catalysts mainly focused on all solution-state derived materials, this review aims at covering the traditional sol-gel process for the “one-pot” synthesis of supported metal and mixed oxide systems. The emphasis will be on the physics and chemistry aspects of the polycondensation of alkoxides with different hydrolysis rates to obtain a homogeneous dispersion of the precursors. In a two components system where the minor component is not a forming network, the metal precursors do not directly participate in the sol-gel chemistry. However, the extent of interaction with the growing gel can be still manipulated within the sol-gel process to obtain an efficient immobilization of the active phase in the host matrix. Some recommendation to tailor the specific surface area still keeping the sol→gel transition will be given to the reader. The different type of chemistry that may be considered under the heading of “sol-gel” will be briefly described. Our intended audience is researchers that are working on the catalytic process who may benefit from knowing how to exploit the versatility of the “traditional” sol gel method.

## 2. Sol-Gel Chemistry

### 2.1. Fundamental Features

Sol-gel chemistry offers a flexible approach to preparing advanced materials, including glass, ceramics, and organic- inorganic hybrids using colloidal solutions (sol) as starting materials [15]. Different configurations (monoliths, coatings, foams, and fibers) can be produced without powder intermediates or the use of expensive processing technologies, such as vacuum methods [15,16]. The “sol-gel” originated from the hydrolysis and condensation of metal alkoxides. The first metal alkoxide was prepared from silicon tetrachloride (SiCl_4_) and alcohol by Ebelman [26], who observed gelation upon exposure of SiCl_4_ to the atmosphere. Despite this, for many years these materials did not arouse an applicative interest. It was not until 1930 that Geffcken [17] discovered that alkoxides could be used in the preparation of oxide films. This process was later fully accepted and developed by the “Schott glass company” in Germany, as illustrated by an excellent review of Schroeder [27].

Inorganic gels from aqueous salt solutions have been studied for a long time. Graham [28] demonstrated that water in silica gel could be exchanged with organic solvents, which supported the theory that the gel is made of a solid network with continuous porosity. This definition was widely accepted in 1930 after a thorough work by Hurd [29], who showed that silica gels had to be formed by a silicon polymer skeleton containing a liquid phase.

The sol-gel technique allows for preparing complex inorganic materials, such as ternary and quaternary oxides, with a homogeneous distribution of components on the atomic scale through a technology of low temperature synthesis and with full control of the finite product microstructure.

**Figure 1:**
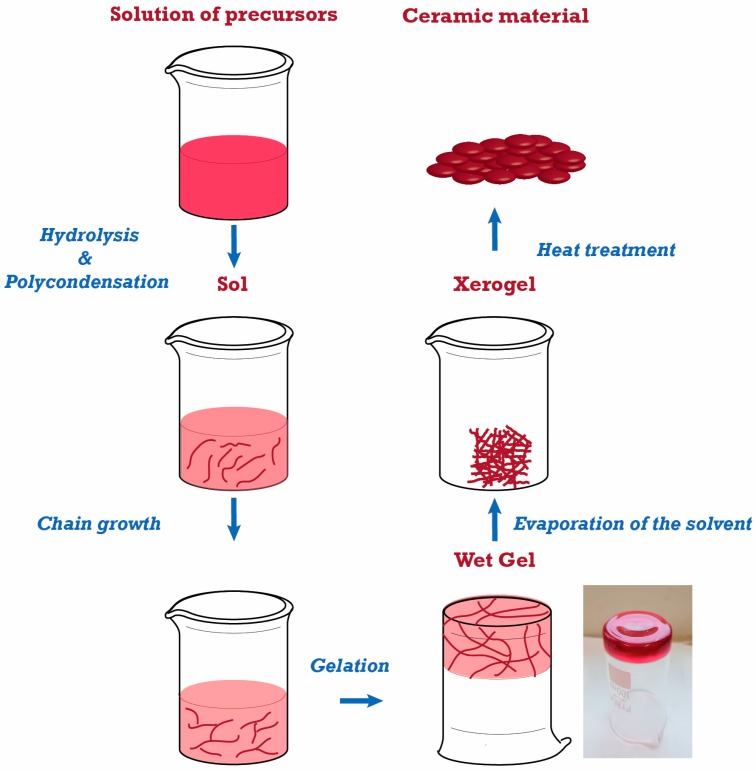
Schematic representation of sol-gel process.

Figure 1 shows the schematic diagram of sol-gel method that can be summarized, as follows:

- Preparation of the solution of precursors.
- Hydrolysis and partial condensation of alkoxides to form a “sol”.
- Formation of the gel via polycondensation of hydrolyzed precursors.
- Drying. The gel forms a dense “xerogel” via collapse of the porous network caused by the evaporation of the solvent (or an aerogel for example through supercritical drying).
- Calcination to obtain mechanically stable materials.

Since chemical reactions continue long after the gel formation, causing changes in the composition, structure, and properties of the gel, an aging step may be added.

The process illustrated in Figure 1 is by no means limiting or exhaustive. Depending on the specific application, the different stages can be extended, altered, or manipulated to respond to different needs. The parameters that could be controlled in the sol-gel method includes (1) concentration and type of precursor, (2) nature of solvent, (3) pH of the solution, (4) type and concentration of additives (catalysts, surfactants, structure directing agents), (5) pre- and post-heat treatment of the materials, and (6) aging time.

### 2.2. Gelation: Hydrolysis and Polycondensation

The gelation process is the result of the hydrolysis and condensation reactions; therefore, the understanding of the reaction mechanisms is the key to mastering sol-gel chemistry.

**Scheme 1:**
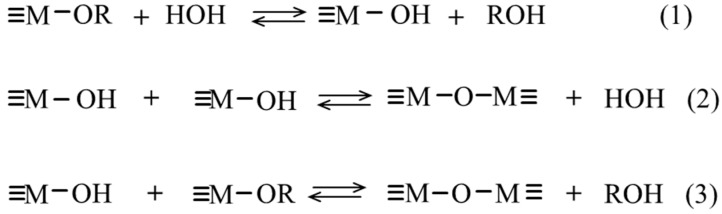
The three main reaction steps occurring in the sol-gel process of an alkoxide M(OR)_4_, (1) hydrolysis of an alkoxy group (2) condensation of two -OH bearing species; and, (3) mixed condensation of a –OH group and an alkoxy group bearing species.

In the hydrolysis reaction, a nucleophilic substitution mechanism is hypothesized, which results in the replacement of an alkoxy group with a hydroxyl (Scheme 1, reaction 1).

When considering an alkoxide of the element M, M(OR)_4_, where R is the alkyl group. The nucleophilic addition of a water molecule is followed by a proton transfer making alcohol (ROH) a better leaving group [15,16,17].

The thermodynamics of this process is therefore governed by the nucleophilicity of the incoming group and by the δ^+^ of charge on the element M. The latter depends on the electronegativity of the element, on the electron donating capacities of the OR group, and on the stability of the leaving group. The reaction rate, on the other hand, increases with the increase in the difference (N-z), where N and z, respectively, indicate the coordination number and the charge of the element M [15]. Another factor influencing reaction kinetics is the complexity of the alkyl chain of the alkoxide (steric encumbrance), in accordance with a nucleophilic substitution mechanism. In this scenario, the behavior of transition metal alkoxide is strongly different from the most common silicon alkoxide (N = z), where neither alcohol association nor oligomerization is observed [15].

In parallel with the hydrolysis reaction, the polycondensation reaction occurs: the partially hydrolyzed alkoxide molecules may either react with another OH– bearing species by removing water (Scheme 1, reaction 2) or react with alkoxy group producing an alcohol molecule (Scheme 1, reaction 3).

The hydrolysis and polycondensation reactions lead to the formation of clusters that bind to each other to form a single three-dimensional polymeric network called gel, at which the viscosity is observed to increases abruptly [15].

In the synthesis of metal oxides, due to the high value of electronegativity of the oxygen with respect to the metal, the M–O–C bonds are, as a rule, highly polarized and the rates of hydrolysis are high. In the case of non-metal alkoxides (e.g., Si, P, Ge), the rates of hydrolysis are slower.

In multicomponent systems (mixed oxide preparation) the different rate of hydrolysis of the precursors can cause the gelation to take place at different times, through homo-condensation reactions rather than hetero-condensation, which leads to phase separation phenomena.

This problem can be overcome by modification of the hydrolysis rate of the most reactive precursor with the use of reaction inhibiting agents (e.g., chelating agents) and by enhancing the hydrolysis rate of the less reactive precursor through a catalyzed (acidic or basic) pre-hydrolysis. The use of double alkoxides with defined stoichiometry is also an alternative option [15,16,17]. The gel properties, and in turn the properties of the material at all subsequent processing steps, can be tuned through the factors affecting the hydrolysis and condensation reactions. It is the control of these sol-gel parameters that renders the sol-gel technique favored in comparison with other preparation methods.

The definition of the gel is still controversial, and we want to remember the more philosophical definition by Nijenhuis: “A gel is a gel, as long as one cannot prove that it is not a gel” [30].

From the lab point of view, gel time can be considered as the time that is required for the solution to undergo a significant viscosity increase during the sol-to-gel transition, such that we can no longer maintain a vortex in the solution with a magnetic stir bar.

**Figure 2:**
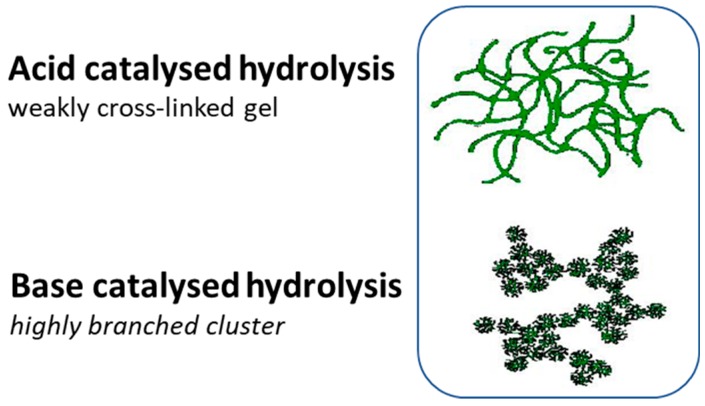
Structure of the gel from acid and basic catalyzed hydrolysis.

Its structure is strongly dependent on the water content in the system and on the catalysis nature. Generally, the silicon dioxide that was obtained starting from acidic “sol” has a network consisting of linear polymers with a low density of crossed bonds; from basic solutions, instead, highly branched clusters are obtained, as in Figure 2. This is the result of the competitive effect of the hydrolysis and condensation reactions whose rates are pH dependent. The reactivity of the silicon alkoxides, Si(OR)_4_, can be also governed by the choice of the alkoxy groups, since the condensation and hydrolysis rates are determined by steric hindrance factors [31,32,33].

### 2.3. Precursors (M and -OR)

The most commonly used precursors in the sol-gel method are those alkoxides, M*_x_*(OR)*_y_*, which have properties that are useful in the chemical control of oxide synthesis. It is possible to choose R from an almost infinite number of alkyl groups according to the desired reactivity. Furthermore, a vast array of alkoxide precursors is available or synthesizable. Mixed alkoxides are a further tool in the control of the stoichiometry and homogeneity of the final products.

A comparison between the chemical behavior of the alkoxides of the non-metallic elements and those of the metallic elements allows for highlighting large differences in the nucleophile reactions, such as those of hydrolysis and polycondensation.

The lower electronegativity values of transition metals and the possibility of expanding their coordination sphere justify the greater reactivity in the hydrolysis reaction in the case where M is a transition metal with respect to the case in which M = Si, P, Ge. In the former case, the synthesis of M*_x_*O*_y_* requires severe conditions in order to prepare gels rather than precipitates. The inductive effect of the R-group also impacts on the stability of the alkoxy groups. These factors affect the relative rates of hydrolysis and condensation, and thus the degree of oligomerization or polymerization. Finally, physical factors, such as volatility and viscosity, drive the choice of an appropriate alkoxides for sol-gel chemistry [14].

A quite large family of organically modified silicon alkoxide, R’*_x_*Si(OR)_4−*x*_, whose properties depends on the nature of R’ promoted the development of a new class of organic-inorganic hybrid materials with unique combinations of properties [34,35].

### 2.4. Converting A Wet Gel into A Dry Solid

The wet gel that was obtained from the reactions of hydrolysis and the polycondensation of the alkoxide is subsequently subjected to a drying process and an appropriate heat treatment to obtain the material with the required features.

In the drying process, the wet gel is heated at temperature of about 100 °C to allow desorption of water and alcohol that are physically bound, as seen in Figure 1. The transformation of wet gel into a dry gel by a simple evaporation leads to a deformation of its primitive porous backbone and it is often accompanied by the formation of “cracks” [15,16,17]. The driving forces for shrinkage include chemical effects, such as condensation reaction, and physical effects, such as capillary pressure. If the pressure in the liquid were uniform, the lattice would be uniformly compressed and there would be no tendency to fracture. The low permeability of the gel, on the other hand, generates a pressure gradient, the difference in the contraction speed between inside and outside the gel is responsible for the formation of cracks.

The product of this uncontrolled drying is called xerogel and, in the absence of structure directing agent, it is characterized by disordered porosity.

Warping and cracking phenomena can be cushioned by aging the gel. The chemical reactions that lead to gelation are shown to continue long after the formation of the wet gel, causing changes in the physico-chemical properties of the gel. The formation of new crosslinks produces shrinkage (syneresis) and increases the modulus and viscosity of the gel, so that aging reduces the subsequent shrinkage during drying [15,16].

Aerogel are obtained from gels in which the pore liquid is replaced by air with moderate shrinkage of the matrix. This is commonly obtained at the supercritical drying (SCD) condition, leading to minimal impact on the porous structure. It is also possible to achieve high levels of porosity through freeze drying, this results in a cryogel, the porosity of which is usually between xerogel and aerogel [36].

The subsequent heat treatment depends on the type of product desired. Generally, the dry gel is heated to temperatures in the range of 300–500 °C to remove residual organics. Calcination often results in more mechanically stable materials, but sintering can cause the density of the materials to increase and the pore volume and surface area to decrease. 

## 3. Sol-Gel Chemistry: The Evolution and New Perspectives

Since its discovery, sol-gel chemistry has been subjected to continuous development, grabbing the attention of a wide scientific community. The impressive rapid development of new synthesis and procedures exploiting different chemistries has funneled all solution-state precursors in the sol-gel world. Although the present review is focused on the “traditional” sol-gel method, we want to remind the reader of the main developments by reporting the most interesting advances.

### 3.1. Sol-Gel Routes for Bio-Catalysts

Recently, the increasing demand of biomaterials has pushed the sol-gel procedure in the biomedical science field. The liquid phase reactions of silicon alkoxides, which are easily controlled through the synthesis parameters, and the possibilities to tailor the physical and chemical properties of the silica network, fostered the design of specific routes for the entrapment of biomolecules or cells [37]. The encapsulation of heat-sensitive and fragile biomolecules is facilitated by the low temperature processing, spurring the development a new generation of bio-catalysts. Alkylated precursors are used to adjust the hydrophilic- hydrophobic balance of the reaction environment, so as to modulate the proteins-matrix interactions and likely promote the access of the substrates to the enzymes. The considerable shrinkage due to the evaporation of solvents, causing increases steric compression and diffusional limitations, has been limited by means of new desiccation protocols, such as lyophilizing processes [38]. A further issue in the sol-gel entrapment of biomolecules is the presence of alcohol in the sol, which can interfere with the activity of the biomolecules. To circumvent this problem, some alcohol-free sol-gel routes were designed using either poly(glyceryl silicate) or sodium silicate as precursors [39,40]. Alternatively, other oxide-based matrices could be explored: TiO_2_, Al_2_O_3_, and ZrO_2_.

### 3.2. Nonhydrolytic Sol-Gel Chemistry

A fascinating route that has its greatest asset in the production of dispersible metal oxide nanoparticles is the non-hydrolytic sol-gel. While no hydrolysis reaction is possible due to the absence of water, the type of condensation rules the formation of metal-oxygen bond. Different solvent can be used to supply the oxygen that is required for the formation of metal oxide (alcohols, ketones, aldehydes, esters), or it can come from the organic part of metal precursors [14,41]. The rates of the reactions involved are slower when compared to the hydrolytic sol-gel approach, resulting in an easier control of the chemical and physical properties of the metal oxide [41]. This method can be particularly valuable in the case of mixed oxide systems, where homogeneity is limited by the presence of water. As discussed in Section 5.1, water has a detrimental effect on the preparation of silicophosphate materials since promotes the hydrolysis of Si–O–P bond leading to low homogeneity gel. Silicophosphate with high degree of homogeneity were prepared by means of ester elimination procedure [42,43]. The attainment of mesoporous metal oxide without an expensive and complex procedure is a remarkable aspect with a great impact on the heterogeneous catalysis. The most relevant results were obtained for silica based mixed oxide, SiO_2_–MO*_n_* systems (M = Ti, Zr, Al) [44].

### 3.3. Modified Pechini Method

The Pechini method owes its name to the author who developed this sol-gel derived synthesis that was patented in 1967 [45].

The chemistry behind this method is that of metal complexes and it is used to prepare bulk materials, nano-crystalline powders, and thin films. As chelating agent of the metal centre, the cheap and readily available citric acid was originally employed. The procedure involves the preparation of a stable aqueous solution of the metal salt and the tricarboxylic acid in the presence of ethylene glycol; the pH is the key parameter to control the extent of the cation binding to the citrate and it generally optimized using ammonia, ammonium hydroxide, or other bases. The acidity of the solution is also an important tool in the prevention of the precipitation of individual hydroxides when several metals are used. The covalent network results from polyesterification between citrate and ethylene glycol. [14,46]. The decomposition or combustion of the organics leads to the ceramic phase. The Pechini method was further developed, replacing the citric acid and the ethylene glycol with other carboxylic acid and polyols, respectively. The great advantage of this method is the cross-linked polymer hinders the degree of homogeneity and purity that were obtained in the preparation of mixed oxides, since the segregations of the cations. Quaternary oxide, like the YBCO superconductor, were prepared as single phase with a modified Pechini method, the replacement of the citric acid with EDTA resulted in effectively to limiting the occurrence of BaCO_3_ secondary phase [47].

## 4. Porosity: The Role of Water/TEOS Ratio

As discussed above, there are numerous parameters that are involved in the sol-gel technique with an important influence on textural and structural properties of the synthesized material. Among them, water is a key parameter governing the sol to gel transition and the gel time. As expected from reaction 1 Scheme 1, the stoichiometric value of water to alkoxide ratio (*R*_w_) is 4, it means that the hydrolysis of a tetravalent alkoxide M(OR)_4_, like the tetraethylorthosilicate (TEOS), Si(OC_2_H_5_)_4_, needs four water molecules to be complete. The increase in the amount of water strongly influences the hydrolysis and condensation kinetics: the dilution effect for *R*_w_ > 5 changes the hydrolysis and the condensation rate in the direction, which leads to longer gelation time [48,49,50].

The effect of water on the porous structure of the xerogel has been long debated, since it depends from the structure of the particles growing from the sol. Yoldas stated that a higher *R*_w_ promotes the formation of higher ratio of bridging to nonbridging oxygens, thus yielding a more polymerized and more branched structure [51]. This is confirmed by the viscosity measurements that were presented by Sakka and Kamiya [52]. They reported that, at a constant molar ratio HCl to TEOS of 0.01, linear polymers are formed for *R*_w_ < 4 and branched polymers for *R*_w_ > 4.

We report a set of experiments made in our lab on the effect of H_2_O/TEOS ratio (*R*_w_) to provide recipes for tailor-making silica. Four porous silica samples were prepared by using either a conventional sol-gel route or an alcohol-free method. In the former tetraethoxysilane, Si(OC_2_H_5_)_4_, (TEOS) was hydrolysed at room temperature using water and concentrated hydrochloric acid with the molar ratios: TEOS:EtOH:H_2_O:HCl = 1:4:2:0.01. In the solvent free method, TEOS is hydrolysed at 50 °C with water and the miscibility of the system is guaranteed by hydrolysis products. The values of the H_2_O/TEOS ratios that were explored in the modified sol-gel route are: 5, 10, and 20. For all solutions, the molecular ratio TEOS:HCI was maintained as equal to 1:0.01. The samples will be referred hereafter as SG-*R,* where *R* is the value of *R*_w_. The gel times were much higher in the preparation with ethanol as co-solvent, whereas in the modified series it increases as a function of *R*_w_, Table 1.

**Figure 3:**
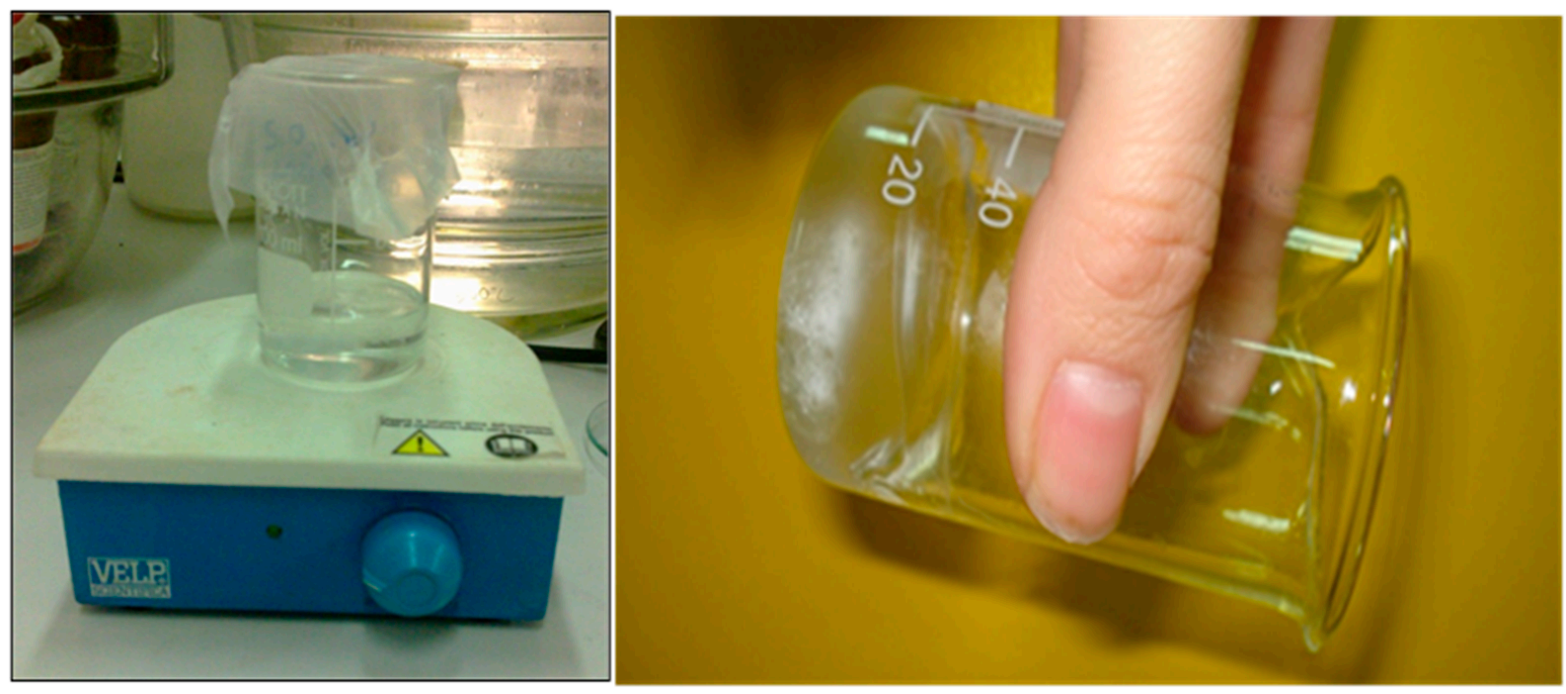
Gel formation of SG-*R*.

Transparent wet gels were obtained for all compositions, as displayed Figure 3.

After aging 72 h at room temperature, the wet gels were dried in air at 110 °C in an electric oven for 12 h. The xerogels were subjected to thermogravimetric/differential thermal analysis (TG/DTA) (data not reported). The DTA curves showed an exothermic peak with a maximum of approximately 300 °C that is ascribable to the combustion of all organic residues. All of the xerogels were heat treated at 400 °C for 1 h and SG-*R* samples were obtained. 

**Figure 4:**
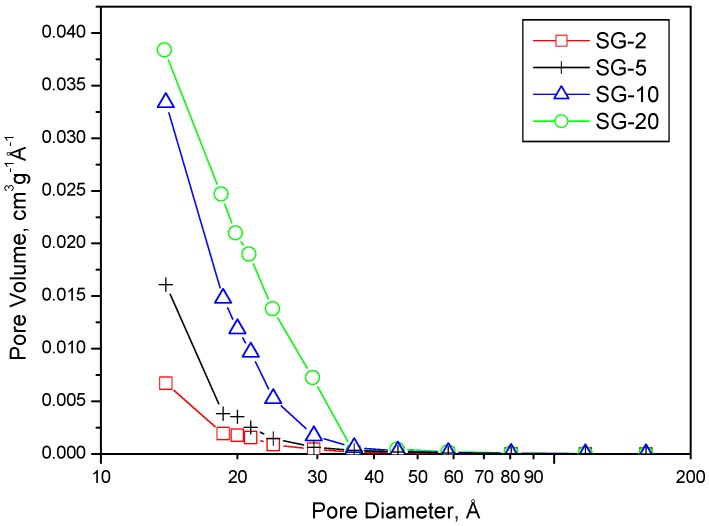
Pore size distribution of SG-*R* sample heat treated at 400 °C for 1 h.

N_2_ adsorption-desorption isotherms of SG-*R* samples has a type I isotherm (not reported), reaching saturation at low *P*/*P*_0_ values, and without hysteresis loop, as typical of a microporous material. The N_2_ volume adsorbed becomes higher while increasing *R*_w_, together with smoother isotherm knee indicating the presence of a wide range of pores diameters. The corresponding values of specific surface areas (SSA), total pore volume (*V*_p_), and micropore volume (*V*_MP_) are reported in Table 1. Pore Size Distributions (PSD) that were obtained by applying Barrett–Joyner–Halenda (BJH) approach to desorption branches of the corresponding isotherms, are reported in Figure 4: in agreement with isotherms shape, the pore volume, and the average pore size increase from SG-2 to SG-20.

The preparation without alcohol, still keeping the homogenous nature of the gel, determines a strong modification of the gel properties. Besides the more manageable gel time, the silicas that were obtained by the modified route (SG-*R* with *R* from 5 to 20) show a substantial modification of the textural properties when compared to the classical preparation that was assisted by the solvent: the surface area increases up to a remarkable value of 705 m^2^ g^−1^, the average pore diameter increases, and then enters the mesopore range. The volume of the micropores becomes negligible when compared to the total pore volume. In conclusion, the ratio H_2_O/TEOS is a powerful tool to modify the textural properties of silica. The syntheses reported in the literature are often referred to a stoichiometric value of water, underestimating the relevance of the H_2_O/TEOS ratio with respect to more complex procedures. By simply changing the value of R, we have obtained a silica gel with a surface area comparable to some zeolites or aerogels. The role of R in the preparation of metal doped silica system to know to which extent it can interfere with the metal-silica interaction and with the metal particles size could be an interesting investigation.

The samples SG-2 and SG-20 were compared and successfully used as adsorbent to remove simazine from polluted waters displaying better performance with respect to commercial zeolites [53,54,55,56,57].

## 5. Supported Metal and Mixed Oxide Systems Synthesized by “Traditional” Sol-Procedures: Some Examples

The preparation of mixed-oxide catalysts and supported metal oxide catalysts commonly involves aqueous-phase methods (ion-exchange, impregnation and deposition–precipitation), which predominate at the industrial scale for environmental and economic reasons [58]. The great interest in the production of new materials with unique properties by sol-gel routes coincides with the growing recognition that the conventional methods of materials processing have inherent limitations in homogeneity due to difficulty in controlling the agglomeration of the active phase, yielding weak binding between active phase and the carrier. Sol-gel preparation allows for the introduction of several components in a single step with a high degree of structural and compositional homogeneity being afforded through the manipulation of the synthesis conditions.

Some examples of catalysts that were prepared by “traditional” sol-gel chemistry enables us to better understand the many resources of this procedure and structure-activity relationship in catalytic materials. The “case studies” reported in this section were selected with the purpose to cover the major current industrial applications of supported and unsupported metal oxide catalysts

### 5.1. SiO_2_–P_2_O_5_

Solid acid catalysts are among the most used in industrial chemical processes [59,60]. They have many advantages over liquid catalysts, such as simplicity of handling, decreasing reactor and plant corrosion problems, and environmentally safe disposal. Solid acid catalysts are largely used in the industrial hydrocarbon chemistry, but there is currently much effort to develop active and low-cost acid catalysts for biomass conversion to produce biofuel [61].

A basic task of the present review is the central role of the synthesis route to consciously tailor the features of the final product to comply with the requirements of the catalytic reaction. The P_2_O_5_–SiO_2_ system was selected as an ad hoc system to describe how to deal with the different reactivity of alkoxide precursors without losing sight of the goal of a homogeneous system.

Silica supported phosphoric acid was used as a solid acid catalyst for olefin oligomerization and hydration [62,63]. Silica-supported phosphorus pentoxide (P_2_O_5_/SiO_2_) was used in several transformations, such as oxidation of sulfides to the corresponding sulfoxides [64], the nitration of aromatic compounds [65], synthesis of nitriles from aldehydes [66], and acylation of amines [67].

Although phosphates and silicates have similar crystalline structures, which consist of tetrahedral units [PO_4_]^3−^ and [SiO_4_]^4−^, respectively, the chemistry of P (V) and Si (IV) solutions is extremely different.

For this reason, when viewed from the perspective of achieving a homogeneous composition, the sol-gel chemistry of the phosphosilicate system appears to be a tricky issue. The most commonly used precursor in the synthesis of silicates from sol-gel systems is tetraethoxysilane Si(OC_2_H_5_)_4_ (TEOS). Since silicon has low electrophilicity and a stable coordination number, the reaction rates are quite slow, so it is necessary to catalyze the hydrolysis reactions of the TEOS with an acid or a base [15,16,17].

The choice of the phosphorus molecular precursor plays a fundamental role in determining the characteristics of the final product, such as the extent of copolymerization between silicate and phosphate units, the type of crystalline phases and their relative amount, the actual phosphorus content, and the textural and surface properties [68].

Livage et al. [69] compared two different molecular precursors, PO(OEt)_3_ (triethilphosphate, TEP) and orthophosphoric acid, PO(OH)_3_. Using Magic Angle Spinning (MAS)-Nuclear Magnetic Resonance (NMR) on ^31^P, they observe a partial hydrolysis of TEP only when the alkoxide was heated under reflux for six weeks in the presence of an excess of water and sulfuric acid as a catalyst. On the other hand, orthophosphoric acid reacted too fast when compared to tetraethoxysilane, leading to precipitation rather than gelation. More useful precursors can be obtained by dissolving P_2_O_5_ in alcohol to form different PO(OH)_3−*x*_(OR)*_x_* species that have an intermediate reactivity between TEP and phosphoric acid, or by using POCl_3_ phosphorus oxychloride.

**Figure 5:**
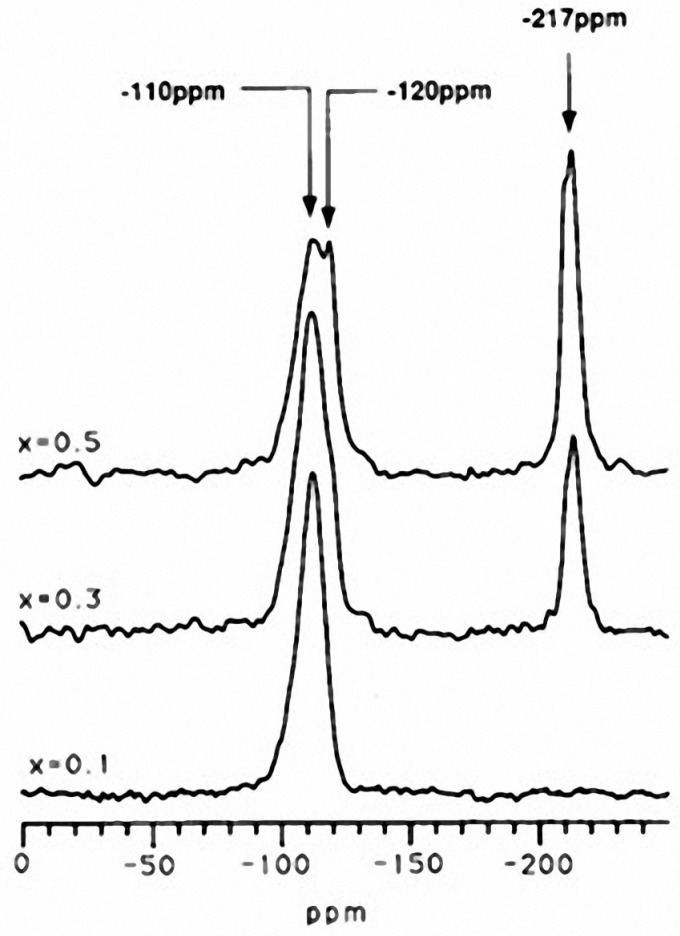
^29^Si MAS-NMR spectra for H_3_PO_4_-prepared gels heat treated at 200 °C for 10 h. Adapted with permission from [70]; Copyright 1992 Copyright Elsevier.

Szu et al. [70] prepared a series of gels in the SiO_2_–P_2_O_5_ system using orthophosphoric acid, triethylphosphate, and trimethylphosphite as phosphorus precursors. Through nuclear magnetic resonance on ^31^P and ^29^Si, they showed that the copolymerization of silicon and phosphorus tetrahedrons only occurred for prolonged heat treatments at temperatures of about 200 °C. The peak at about −120 ppm in the ^29^Si MAS-NMR spectrum, Figure 5, was assigned to the Q_4_ unit that is associated with P (i.e., Si–O–P).

The same authors found that the type of precursor of P_2_O_5_ greatly influences the crystallization behaviour. Gels that were prepared with TEP were amorphous after heat treatments at 800 °C for 10 h, whereas the ones that were obtained from the trimethylphosphite are crystalline at much lower temperature. The gels prepared with H_3_PO_4_ crystallize partially only if the content in moles of P_2_O_5_ exceeds 10%, forming Si_5_O(PO_4_)_6_ where part of the silicon assumes VI coordination.

Schrotter et al. [71] followed the transformations occurring in a mixture of tetraethoxysilane and a phosphorus alkoxide [P(OEt)_3_ or (OEt)_2_P–O–P(OEt)_2_ or PO(OEt)_3_] by ^1^H, ^13^C ^29^Si, and ^31^P liquid and solid-state NMR, Infrared, and Raman spectroscopies. This study unveiled different behaviors towards hydrolysis for these three different phosphates and phosphites. The triethylphosphate [P(OEt)_3_] reacted almost instantaneously with water to form intermediate species, HPO(OEt)_2_, which slowly transform into HPO(OH)(OEt). After 21 days, the phosphorus was present in solution as orthophosphoric acid. The same species are formed faster in the sols containing (OEt)_2_P–O–P(OEt)_2_ by breaking the P–O–P bond and subsequent hydrolysis. The TEP hydrolyzes very slowly, after 10 months, the main constituents of the solution were still the pristine alkoxides. In all three cases, the P–O–P and Si–O–P bonds were only present in solution in very small quantities.

It could be concluded that the mechanism of gelation of phosphosilicate solutions involves the formation of silicon “clusters” entrapping phosphorus monomeric species (or dimers). The type of phosphorus precursor used can, however, influence the kinetics of the hydrolysis and polycondensation reactions of the TEOS and consequently the gelling times.

More recently, D’Apuzzo et al. [72] reported the sol-gel synthesis of phosphosilicate in bulk and in thin films shape using a modified POCl_3_ precursor. In approaching the hydrolysis rate of the tetraethoxysilane, POCl_3_ was mixed with anhydrous ethanol in molar ratio 1:6, allowing for the formation of POCl_3−*x*_(OEt)*_x_*. The partial substitution of chlorine atoms by OEt groups reduces the positive charge of P atom, thus decreasing its reactivity toward water.

**Figure 6:**
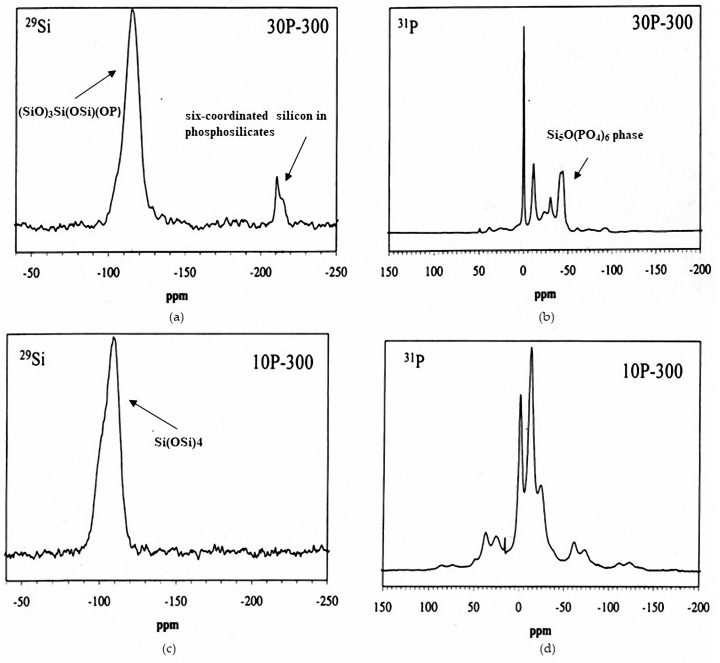
^29^Si and ^31^P MAS-NMR spectra of the gels heat treated at 300 °C containing 30 mol % P_2_O_5_ (30P-300) and 10 mol % P_2_O_5_ (10P-300): (**a**) ^29^Si of 30P-300; (**b**) ^31^P of 30P-300; (**c**) ^29^Si of 10P-300; (**d**) ^31^P of 10P-300. Adapted with permission from [73]; Copyright 2003 Copyright The Royal Society of Chemistry.

Solid state ^29^Si and ^31^P MAS-NMR was used to characterize the gels, 10P_2_O_5_·90SiO_2_, and 30P_2_O_5_·70SiO_2_, and to investigate their microstructural changes during thermal treatments [73]. Both of the dried gels showed similar structures that were formed by orthophosphoric acid and, in very little amount, by pyrophosphoric acid that is embedded in a siloxanes framework. The formation of the P–O–Si bonds, as well as of silicon hexa-coordinated, was detected in the gel with the 30 mol % of P_2_O_5_ heat treated at 300°C. At the same temperature, a more polymerizate siloxane network was observed for lower phosphorus content, as seen in Figure 6. The different heating behaviour of the studied gels was explained with a different distribution on the atomic scale of phosphorus atoms in the pore of the siloxane framework [73]. 

The authors [74,75] also performed on 10P_2_O_5_·90SiO_2_ and 30P_2_O_5_·70SiO_2_ gels solid state ^1^H MAS-NMR with ^1^H T_1_ and T1ρ relaxion times, as well as low temperature ^1^H solid echo NMR. The gels were found to be heterogeneous on a nanometric scale and formed by phosphorous domains having different sizes. Unlike 10P_2_O_5_·90SiO_2_ gel, the sample with higher phosphorus content showed uniform distribution of small domains, giving rise to dynamic process that are related to chemical exchange between the POH groups.

A comparative structural investigation by Raman, NMR, XRF, and XPS spectroscopies on phosphosilicate gels was also carried out by Todan et al. [76] to shed light on the mechanism of P–O–Si bonds formation. They confirmed the inadequacy of the triethylphosphate as precursors in the sol-gel preparation, whereas partially hydrolysed phosphorus units were detected for triethylphosphite, P(OC_2_H_5_)_3_, precursors, indicating a certain degree of reactivity toward water. It is worth stressing that the type of silicon and phosphorous units forming network is crucial in determining the final composition and structure of the gel derived materials.

### 5.2. Co–SiO_2_

Cobalt that is entrapped in a tailored matrix represents one of the most fascinating materials among the transition metal-based systems in name of its activity in many catalytic processes [77,78,79,80,81,82,83]. Recently, Co has emerged as the most versatile non-noble metal in the development of H_2_-and O_2_-evolving catalysts in the water splitting reaction [84,85,86].

The stable oxidation state of cobalt in solution is +2 and cobalt nitrate or acetate are generally used as precursors. Embedded in the support, Co^2+^ is prone to oxidation and Co_3_O_4_ is the stable oxide at room temperature. It can be depicted as a [Co^2+^Co_2_^3+^O_4_] mixed valence oxide with a cubic spinel structure, where Co^2+^ and Co^3+^ ions occupy one-eighth of tetrahedral (Td) sites and one-half of octahedral (Od) sites, respectively. Above 950 °C, Co_3_O_4_ is reduced to (stoichiometric) CoO. High valence Co species, i.e., those occurring in the cubic spinel Co_3_O_4_, are considered as the precursor of the metallic catalytic species (Co^0^), in virtue of their low temperature reducibility. On the other hand, the segregation of Co_3_O_4_ is symptomatic of a low dispersion of Co species, which in turn is detrimental for catalytic activity [87,88,89]. 

The formation of Co_3_O_4_ is indeed strictly affected by the selected support and by the preparation method. SiO_2_ is one of the most versatile supports, because SiO_2_ has excellent chemical and thermal stability, fair accessibility and porosity, and because organic groups can be anchored at the surface, providing stable catalytic centers. 

Several recent studies on cobalt-silica systems have shown that the preparation method and the structure of the silica matrix has a marked influence on the type and dispersion of cobalt oxide species, and thus on the properties of the derived catalysts [2,90,91,92,93,94,95,96]. For this reason, a big effort has been spent in tailoring the materials’ features through the variation of the synthesis parameters. By means of a flame pyrolysis (FP) technique, Co–silica catalysts were prepared as possible substitutes for Ni-based catalysts, very active for the Ethanol Steam Reforming reaction, but showing poor stability towards coke formation when operating at relatively low temperature [90,97]. The best results were achieved with 10 wt % Co/SiO_2_, which led to higher activity, good C balance, and low CO/CO_2_ ratio with respect to Ni catalysts. The authors suggested that the strong metal-support interaction, the absence of any cobalt segregation, and the dispersion on the catalyst surface of reducible cobalt species were the determining factors in the activity at higher temperature [90,97]. However, the catalytic tests at 500 °C are disappointing and the best chemical formulation operating at such temperature it is still lacking. The effect of cobalt loading on Co–SiO_2_ that is prepared by FP is not explored and the 10 wt % Co is the single composition reported.

The cobalt-silica interaction, if not properly modulated, can favor the formation of a cobalt silicate phase. According to Puskas et al. [92], this is a further issue in the preparation of active Fischer Tropsch catalyst, because it requires impractically high temperature being unsafe for the surface properties of metallic cobalt species.

The use of periodic mesoporous silicas as supports of cobalt based catalyst is becoming increasingly popular due to the large surface area and uniform mesoporous channel, which increases the real potential in catalysis. Essentially, these systems can be obtained by direct synthesis (generally referred as one-pot method) or by the classical impregnation procedure.

Vizcaíno et al. [96] prepared SBA-15 supported Co catalysts that were modified by the addition of Mg and Ca to study the different effect of these promoters on the catalytic performance. The addition of alkali-earth metals limited the fraction of Co_3_O_4_ promoting strongest metal-support interaction, but it was not effective in maintaining enough active Co^0^ species.

Martínez et al. [93] prepared Co/SBA-15 by impregnation with solution of different cobalt salts. The nature of the cobalt phase resulted in being closely related to cobalt precursors: Co_3_O_4_ was the only crystalline phase in all catalyst prepared from cobalt nitrate, while very high cobalt dispersion was observed in the case of cobalt acetate and acetylacetonate precursors. However, the absence of Co_3_O_4_ oxide phase was generally associated to low cobalt content and the use of precursors different from cobalt nitrate [93]. The introduction of high cobalt content in mesoporous silicas by the one-pot procedure is still challenging, effort more pronounced at acid pH, since the acidity environment that is used for the preparation of well-ordered meso-structure promotes the solubility of the cobalt species and their effective introduction in the framework [94,98,99].

Even though we have intentionally limited the discussion to some representative works, the general principle is the lack of fundamental studies on how “to play” with Co redox chemistry, to comply with the need of a fine balance among Co reducibility, dispersion, and reactivity.

Ionic and non-ionic surfactants are successfully used in the supramolecular templating approach, since the development of the periodic mesoporous silicas in 1992 at Mobil oil company. The mechanism of self-assembly is driven by the concentration of the structure directing agent, and it leads to a composite mesostructured during the condensation of the silica network. These reactions form highly structured materials with different arrangement of mesopores.

The development of porous materials with large specific surface is an area of extensive research that can count on a high number of papers. In the present review, we want to draw the reader’s attention to an alternative role of the surfactants below the critical micelle concentration (CMC) point.

**Figure 7:**
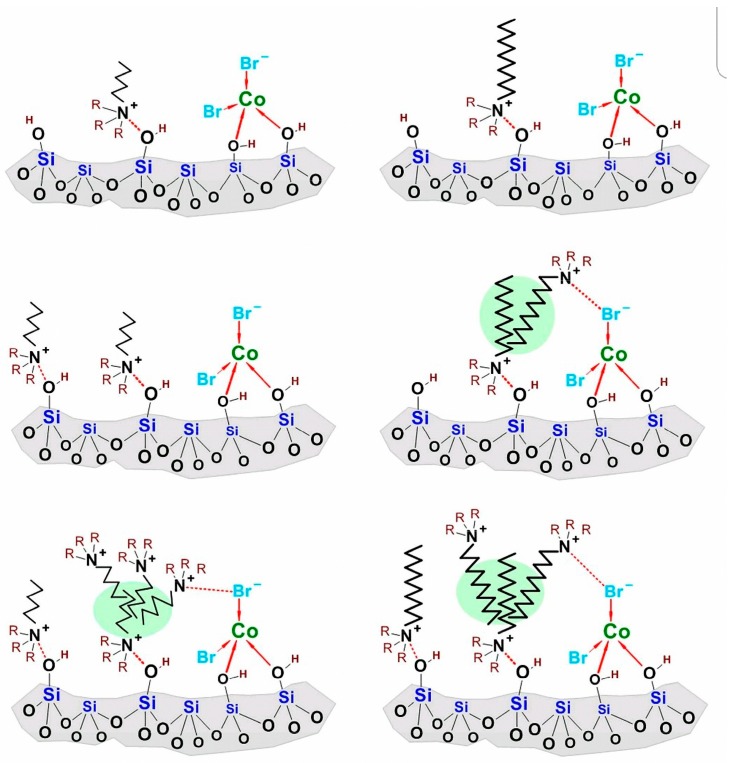
Inhibition of cobalt oxidation. The LEFT sequence represents small surfactants (tetraethyl ammonium bromide and triethyl hexyl ammonium bromides). The RIGHT sequence represents surfactant with higher number of carbon (dodecyl trimethyl ammonium bromide). The concentration of surfactant increases from the top to the bottom. Local aggregation is evidenced by the green area. Reprinted with permission from [100]; Copyright 2014 Copyright The Royal Society of Chemistry.

Recently, Olguin et al. [100] unveiled the important counter effect of the template agent on the embedded cobalt in the sol-gel silica matrix. By using a short cationic surfactant, 3 hexyl triethyl ammonium bromide, the cobalt oxidation state was substantially modified as a function of the template agent amount. They reported an enhanced formation of Co_3_O_4_ at low concentration of surfactant promoted by CoBr*_x_*^2−*x*^ species, whereas the reversed effect at high loading was related to the increased interactions between the CoBr*_x_*^2−*x*^ species, the surfactant head groups, and the silica surface [100]. Maintaining the cobalt precursors content unchanged, the oxidation state of cobalt was tailored by varying the concentration and carbon number of quaternary ammonium surfactants, as seen in Figure 7.

N_2_ adsorption/desorption measurements allowed for evidencing the tailoring ability of these surfactants. The nitrogen adsorption increases with the surfactant concentration, while a type I isotherm was observed for all of the composition. Therefore, the surface area can be altered with the surfactant content still preserving the microporous structure [100].

**Figure 8:**
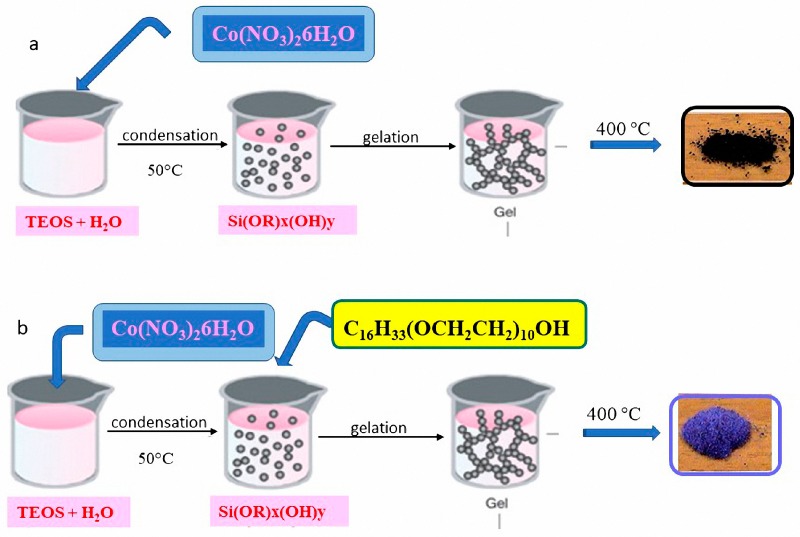
Schematic representation of the synthesis of CoO*_x_*–SiO_2_ by (**a**) hydrolytic alkoxide sol-gel route and (**b**) non-ionic surfactant assisted sol-gel route.

To reveal the versatility of the sol-gel method, while maintaining the formation of a monolithic gel, we designed a modified hydrolytic alkoxide sol-gel route (Figure 8a) and a non-ionic surfactant assisted sol-gel route, as seen in Figure 8b [90]. The aim was to explore to what extent the traditional sol-gel is effective in driving the formation of specific Co phase(s), finally tailoring the catalytic features [90].

**Figure 9:**
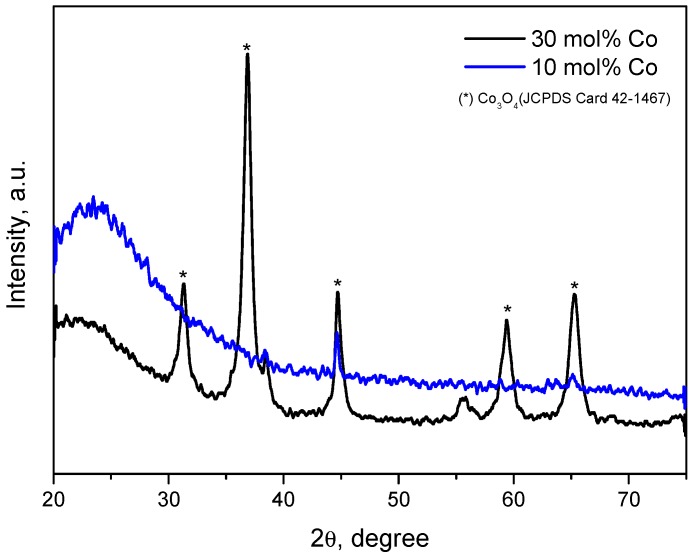
X-Ray Powder Diffraction (XRPD) patterns of samples CoO*_x_*-SiO_2_ prepared by the hydrolytic alkoxide sol-gel route with 10 and 30 mol % of cobalt. Adapted with permission from [90].; Copyright 2018 Springer Nature.

TEOS, as with all the alkoxysilanes, is immiscible in water and a mutual solvent is generally used to homogenize the solution. In the pathway of Figure 8, tetraethoxysilane (TEOS) was hydrolyzed without any alcoholic solvent and the alcohol generated as hydrolysis by-product was used to set the system in the miscibility region. This method facilitated the introduction of hydro-soluble inorganic precursors into the synthesis medium, allowing for the preparation of homogeneous gels with cobalt loading up to 30 mol %. Moreover, it led to a decrease in the gelation time with respect to the conventional alcoholic sol-gel procedure [77]. The gelation was the result of polycondensation reactions involving partially hydrolysed Si(OR)*_x_*(OH)*_y_* oligomers and the wet gels were formed by a siloxanes network, in which the Co species are physically “trapped”. Tetrahedral Co^2+^ complexes strongly bonded to the siloxane framework were observed in samples with lower cobalt content, whereas the formation of Co_3_O_4_ nanocrystals was evident in samples with 30 mol % of cobalt heat treated at 400 °C, as seen in Figure 9.

Fisher Tropsch reaction enables the transformation of syngas to high quality liquid fuels. As stated previously, the dispersion and the reducibility of the cobalt species are crucial features in optimizing the catalytic performance. An extensive redox characterization was carried out by Bagnasco at al. [101] by means of the Temperature Programmed Reduction (TPR) and Temperature Programmed Oxidation (TPO). The hydrolytic alkoxide sol-gel route synthesized different compositions in the system CoO*_x_*–SiO_2_. Three cobalt phases were detected: highly reducible Co_3_O_4_, cobalt silicate reducible at 700–800 °C, and a not reducible fraction. By the H_2_ consumption, the authors managed to calculate the Co distribution (%) as a function of cobalt content and temperature of heat treatment. The close contact of cobalt ions with the growing silica matrix during the hydrolytic alkoxide sol-gel route resulted in a non-negligible drawback in the sample with 10 mol % of cobalt. The not reducible fraction represented the nearly 90 % of the total cobalt loading, making the catalyst unusable; this fraction dropped to 13 % with higher amount of cobalt. The heat treatment of the xerogel at 600 °C did not worsen the redox properties of the CoO*_x_*-SiO_2_ gel with 30 mol % of cobalt. 

High metal loadings are usually required for active transition of non-noble metals catalysts for ethanol steam reforming [97]. However, the big metal particles are commonly responsible for a very insidious coking route. In the surfactant-assisted sol-gel synthesis, the authors explored an unconventional use of a nonionic structure directing agent. A commercially available non-ionic surfactant, Polyoxyethylene (10) cetyl ether (Brij C10), was added to the solution containing partially hydrolyzed Si(OR)*_x_*(OH)*_y_* and [Co(H_2_O)_6_]^2+^ aquo-ions, as seen pathway b Figure 8. The main issue of this approach was to have a molecule acting both as a porogen template, to modify the textural properties, and as an oxygen-rich complexing agent of the metallic species [90]. The selected surfactant/TEOS ratios was selected to guarantee homogeneous sol. The gel formed at room temperature after 48 h. Diffuse Reflectance (DR) UV-Vis spectroscopy allowed for confirming that surfactant deeply interferes in the cobalt chemistry: Co_3_O_4_ formation was successfully hindered by the addition of the Brij C10. The Polyoxyethylene (10) cetyl ether resulted in being effective as an oxygen-rich complexing agent of the metallic species, preventing the formation high valence Co oxide clusters (i.e., Co_3_O_4_), even at 30 mol % of cobalt, as evidenced by the different colour of the heat treated sample, as reflected in Figure 9. The bluish colour is typical of the Co(II) in tetrahedral coordination, whereas the black colour is indicative of Co_3_O_4_ formation. The different cobalt species impact on the morphology of the samples. Field emission scanning electron microscopy (FESEM) highlighted the occurrence of nearly spherical particles with mean size of 30 nm in the sample prepared by the hydrolytic alkoxide sol-gel route [90]. Such particles were not observed in the FESEM picture of the sample that was prepared with the addition of the ionic surfactant. The EDX measurements on five different spots indicated a composition of 29 mol %, i.e., very close to the nominal one [90]. It is likely that the polyoxyethylene (10) cetyl ether might create a reducing environment limiting the oxidation of the Co^2+^. The high degree of solubility of the head groups of poly(ethylene oxide) (PEO) in the silica wall impacts on the porous structure of the gels shifting the pore size distribution toward remarkably high values of surface area and pore size helpful for the catalytic activity [90].

Khodakov et al. [91] highlighted the relevance of the porous structure of catalytic support on the dispersion and reducibility of cobalt species. Both the periodic mesoporous and commercial mesoporous silicas were used as catalytic support with cobalt content of 5 wt %. The Co_3_O_4_ oxide was detected in all samples, while the pore structure of the selected support affected the particle size and so the reduction degree

The surfactant-assisted sol-gel procedure is a fascinating route for the immobilization of Co in the silica matrix at high metal loading tailoring, in both the pore morphology and the metal oxidation state. This synthetic method could be successfully applied to other metal doped silica catalysts, leading to the considerable dispersion of the active phase at high loading, together with advantageous modification of the textural properties tailored according to catalysis needs.

### 5.3. Cu–ZrO_2_

The oil crisis combined with the commitment to reduce the greenhouse gases (GHG) emissions has increased the need of seeking clean and renewable sources of energy. The need to satisfy the demands of new technologies and new approaches to produce and use energy have stimulated great interest in the scientific community. In this scenario, hydrogen is considered as a clean energy carrier that can be used in fuel cells for mobile and stationary applications. Proton exchange membrane fuel cells (PEMFCs) are highly valued due to their high theoretical efficiency and only having steam as byproduct if pure hydrogen is used.

To accrue the full environmental benefits of hydrogen as an energy carrier, low-carbon intensive, low polluting, and lower cost processes for producing hydrogen from renewable energy sources need to be developed [102,103].

Actually, the most attractive solution is the production of high purity hydrogen from biofuels. Hydrogen can be produced by biofuel through different processes (steam reforming (SR), partial oxidation (PO), dry reforming (DR), or autothermal reforming (ATR)) [83]. Among other fuels, methanol is an interesting hydrogen source for its safe handling, low cost, and ease of synthesis from a variety of feedstocks [104,105,106]. Although mostly produced from natural gas, methanol can also be obtained from renewable sources, thus adding no anthropogenic carbon dioxide to the atmosphere [107,108].

Moreover, methanol is recommended as the best source for hydrogen among high energy density liquid fuels, due to the high hydrogen-to-carbon ratio, the lower propensity for soot formation than other hydrocarbons, the relatively low boiling point, and the easy storage [105,106,108].

Although precious metals, such as Pt- and Pd-based catalysts, exhibit prominent activity for OSRM (oxidative steam reforming of methanol); the high cost and scarcity of noble metals severely impede the large-scale commercialization of clean electrochemical energy technologies. As an alternative, OSRM was mainly studied on supported metal Cu and Ni catalysts [104,109]. The presence of both Cu and Ni metals seems to increase the catalytic activity, however Cu promotes the steam reforming reaction, whereas Ni prevalently promotes the decomposition of methanol to CO and H_2_ [109,110]. The more recent studies are mainly addressed to catalysts based on metallic copper dispersed on different oxides, like as SiO_2_, Al_2_O_3_, ZnO, and ZrO_2_ [111,112,113,114]. The nature of the support plays an important role towards product selectivity and the deactivation behaviour of the catalyst. Acidic supports are more prone to deactivation on account of coke formation. With respect to the common used Al_2_O_3_ and SiO_2_, ZrO_2_ is of special interest because of its weak acidity and basicity, its mechanical and thermal stability, its redox, and semiconducting properties [105,106,115,116,117,118,119].

In this context, the authors [105,106,120] reported that sol-gel synthesis is a noteworthy method to properly disperse copper on zirconia support, whereas the formation of copper oxide nanoparticles on zirconia surface is generally observed by using impregnation techniques. 

When considering the three well-defined polymorphs of zirconia, the formation of low temperature meta-stable cubic or tetragonal phase can be promoted by the isomorphic substitution. Cu^2+^ ions that were substituted in the framework sites of Zr^4+^, indicative of high dispersion of dopant metal ion, were detected in Cu–ZrO_2_ that was prepared by traditional sol-gel [121].

Since the nature of the obtained phase governs the physical properties of zirconia, it is important to use a synthesis approach that allows for tailoring the structural properties. 

A recurrent problem in sol-gel preparations is the handling of transition metal alkoxides. Due to the great polarity of the Zr–O bond, they are susceptible to nucleophilic attack and can be hydrolysed by exposing it to air moisture. In addition, transition metals often exhibit several stable coordinations, and when coordinatively unsaturated, they are able to expand their coordination via solvation or alkoxy bridging [15,16,17]. For this reason, the Cu–ZrO_2_ system has been selected to show how to overcome the inconvenience of the fast hydrolysis, preventing undesired precipitation. The most commonly inhibitors are complexing ligands, β-diketones, polyhydroxy acids, or carboxylic acids, which reduce the reactivity by increasing its coordination number and decreasing the number of easily hydrolysable groups [122,123,124,125].

Despite the widespread use of “organically modified metal alkoxides” in sol-gel processes, there are few systematic investigations regarding the inhibitor effect on the metal dopant. In the work of Esposito et al. [105,106], Cu–ZrO_2_ catalysts for the OSRM reaction were prepared by using a conventional hydrolytic alkoxide sol-gel route, acetylacetone (acac) was tested as chelating agent and Cu(NO_3_)_2_·2.5H_2_O as copper precursors. As the matter of fact, the acac plays a double role: it acts as Zr chelating agent and as Cu complexing agent. The content of acetylacetone was varied and a ratio Zr:acac = 1:0.5 was the most suitable in promoting the formation of a polymeric zirconia gel that embeds the copper species [105]. As alternative route, the authors explored the effect of the acetic acid, which possesses conflicting functionalities: it can decrease the reactivity toward hydrolysis acting as bidentate ligand of the Zr alkoxide and it can facilitate the kinetics of hydrolysis through acid catalysis [106]. Moreover, chemical modification of transition metal alkoxide by ligand exchange can interfere with the condensation pathway and with the whole process of sol formation [122,123,124,125,126,127]. The authors investigated the effect of the molar ratio HAc/Zr on gel formation: a rapid reaction with water, with massive precipitation, occurred for an acid to alkoxide ratio lower than 1.6. HAc/Zr equal to two ensured the formation of a stable sol without limiting the gel formation, which is in agreement with the findings of Hayashi et al. [128]. The in-situ source of water coming from the esterification reaction between acetic acid and ethanol accounted for the lower amount of added water with respect to the preparation with acetylacetone [106].

**Figure 10:**
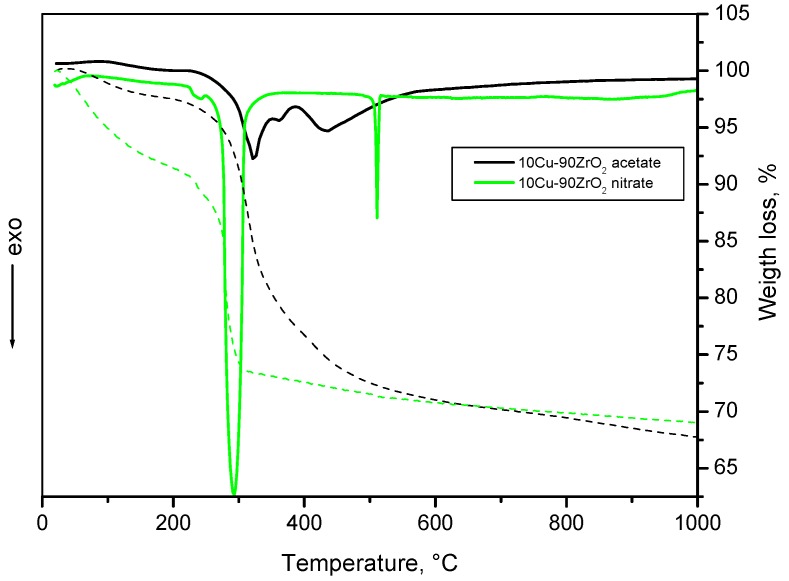
Differential thermal analysis (DTA) (solid line)–thermogravimetric (TG) (dot line) curves of the dried 10Cu–90ZrO_2_ where acetylacetone was tested as chelating agent and copper nitrate as copper precursors (green curves), and 10Cu–90ZrO_2_ obtained by using acetic acid glacial and copper acetate (black curves). Adapted with permission from [105]; Copyright 2010 Elsevier. Adapted with permission from [106]; Copyright 2011 Elsevier.

The thermal and textural properties of ZrO_2_ were appreciably modified by the sol-gel route. The authors [106] observed a delay in the crystallization of zirconia, as shown by thermogravimetric/differential thermal analysis DTA/TG analysis Figure 10, with a consequent modification of the surface area with respect to the pure zirconia that was treated at the same temperature. This effect was explained hypothesizing that Cu^2+^ ions are incorporated into the ZrO_2_ lattice replacing Zr^4+^ ions. The phenomenon, which is observed when the copper nitrate is used as precursor, is hindered in the case of copper acetate, since the limited amount of Cu^2+^. The strong interaction of Cu^2+^ species with ZrO_2_ support led to a remarkable dispersion of metal copper together with a valuable surface area. Considering the amount of copper, the traditional sol-gel method resulted once again advantageous if compared with more conventional methods [129]. The reaction with N_2_O followed by TPR was found to be a valuable method for measuring the dispersion of Cu on ZrO_2_: the sample is firstly pre-reduced in H_2_ flow and, subsequently, adsorptive decomposition of N_2_O is performed for Cu(0) to Cu_2_O oxidation [105,130]. The sample is finally subjected to TPR analysis. The sol-gel procedure that was reported by Esposito et al. [105] led to worthy value of copper dispersion if compared with the more conventional precipitation/impregnation methods [130].

The preparation routes that lead to more homogenous samples often have the drawback of occluding a fraction of the active phase in the bulk of the support, therefore lower value of Cu/Zr can be found on the surface. X-ray photoelectron spectra (XPS) is a valuable method to characterize the surface properties of the catalysts. CO_2_ hydrogenation to methanol was performed on Zn and Ti doped CuO–ZrO_2_ samples prepared by the sol-gel, solid-state reaction, and solution-combustion methods. A comparative investigation by means of XPS and H_2_-TPD (temperature programmed reduction) were able to identify the causes of the difference between the catalytic activities of the samples: mainly the surface of copper content and the H_2_ adsorption capacity [131].

## 6. Conclusions

The utilization of heterogeneous catalysis in most areas of the chemical industry is constantly growing as the environmental and economic pressures drive the research toward clean and selective chemical processes. In this scenario, the development of new catalysts for more efficient and cost-effective process is the main needful objective. An important class of industrial catalysts consists of an active component that is dispersed over a suitable support mostly prepared by post synthesis methods. However, appropriate formulations to promote the desired reaction, prevent undesirable side reactions, and enhance catalysts longevity are still lacking. As described throughout this review, the sol-gel method, as high controllable synthesis, has opened new paths for catalysts preparation. The term ‘sol-gel’ has significantly broadened from the original usage to describe hydrolysis and condensation processes and many newer methods may not involve a clear sol-gel transition. In this work, we have illustrated, with some examples, different ways in which the “traditional” sol-gel can be fruitfully used for the preparation of heterogeneous catalysts. 

## Figures and Tables

**Table 1:** Gelation time (*T*_gel_), Specific Surface Area (SSA), Pore volume (*V*_P_), and Micropore volume (*V*_mp_) of SG-*R* samples.

Samples	*R*_w_	*T*_gel_ (days)	SSA (m^2^ g^−1^)	*V*_p_ (cm^3^ g^−1^)	*V*_mp_ (cm^3^ g^−1^)
SG-2	2	60	410	0.183	0.151
SG-5	5	4	528	0.242	0.186
SG-10	10	9	588	0.285	0.131
SG-20	20	15	705	0.364	0.056
